# Postharvest Drying and Curing Affect Cannabinoid Contents and Microbial Levels in Industrial Hemp (*Cannabis sativa* L.)

**DOI:** 10.3390/plants14030414

**Published:** 2025-01-31

**Authors:** Yousoon Baek, Heather Grab, Chang Chen

**Affiliations:** 1 School of Integrated Plant Science, Horticulture Section, Cornell University, Ithaca, NY 14853, USA; yb273@cornell.edu (Y.B.); heathergrab@psu.edu (H.G.); 2 Department of Food Science, Cornell AgriTech, Cornell University, Geneva, NY 14456, USA

**Keywords:** hemp, drying, curing, cannabinoid, postharvest

## Abstract

Postharvest operations affect the yield and quality of industrial hemp (*Cannabis sativa* L.). This study aimed to investigate the postharvest drying and curing effects on the key quality and safety indicators of cannabinoid-type hemp. Freshly harvested hemp inflorescence of Hempress and Wild Bourbon cultivars were dried by three methods: (1) Hot air drying at 75 °C; (2) Ambient air drying at 25 °C; and (3) Freeze drying. The dried hemp was then cured in sealed glass jars or mylar bags in dark conditions at ambient temperatures. The drying time, overall cannabinoid contents, decarboxylation level, color metrics and total aerobic loads were experimentally determined. Hot air drying can reduce the hemp moisture from 77% to safe-storage level of 6% within 8 h, and achieved up to 2-log reduction in the total yeast and mold counts. The drying time required for ambient air drying and freeze drying were 1 week and 24 h, respectively. Curing led to a 3.3% to 13.6% increase in hemp moisture, while the influence of curing method was not significant. Both drying and curing did not significantly affect the total cannabinoid contents, but resulted in decarboxylation, and reduction in the greenness. The findings suggested that hot air drying followed by glass jar curing is preferred for higher drying efficiency, better preservation of the cannabinoids and microbial safety.

## 1. Introduction

High cannabinoid hemp refers to varieties of *Cannabis sativa* L. plant that are legally defined as having lower than 0.3% tetrahydrocannabinol (THC, one of the major psychoactive compounds), while containing high levels of other cannabinoids, such as cannabidiol (CBD) and cannabigerol (CBG) [1]. This type of hemp is harvested for its inflorescence, where the cannabinoids are predominantly produced and accumulated [2]. In 2022, the United States produced about 6.78 million pounds (~3.08 million kg), which were harvested from 7105 acres, and contributed $179 million USD to the country’s agri-economy [3]. Hemp inflorescences have high moisture content and water activity at harvest, making them vulnerable to chemical and microbial decays, and to pathogenic microorganisms’ growth at the same time [4]. Postharvest loss due to these challenges poses a significant economic cost for producers, emphasizing the importance of good postharvest processing practices, which have not been extensively studied or published.

Drying is a postharvest process that removes moisture from the plant material and has been used to effectively improve the stability of hemp in the industry [5]. Conventionally, hemp is dried indoors within ventilated facilities, either by hanging the whole plant upside down on strings, or wet-trimming then drying the inflorescence on trays. The temperature and relative humidity (RH) of the drying air is usually controlled at around 15 to 20 °C and 50% to 60% RH, respectively. Although these conventional drying methods have the advantage of easy handling, they also suffer from low processing efficiency (drying usually take 1–2 weeks) due to the slow heat and mass transfer rates during the natural convective drying process [6,7]. In particular, when the whole plants are hung and dried, the outer layer of the plants droops down and shrinks first, which wraps around the inner part of the plant, and significantly reduces the drying rates throughout the remaining drying process. This results in moisture non-uniformity in the dried material, degradation of cannabinoids and other bioactive compounds, and rapid growth of spoilage or pathogenic microorganisms in the inner parts of the plant, causing quality and safety concerns and resulting in product revenue loss. In recent years, several attempts have been made to explore the feasibility of emerging drying technologies to efficiently dry hemp. Chen et al. [7] used hot air (HA) at different temperatures to dry hemp flowers and evaluated the impacts of drying conditions on the key cannabinoids and terpenes contents. Oduola et al. [8] studied the influence of infrared (IR) and microwave (MW) drying on the color, cannabinoids, and volatile contents in hemp. Chasiotis et al. [9] investigated the drying kinetics, energy consumption, and cannabinoid contents in hemp inflorescence using time-varying convective drying temperature schemes. Chen et al. [10] showed that sequential IR dry-blanching and HA drying can effectively reduce the microbial levels and inactivate the natural enzymes in the hemp flowers. Meanwhile, freeze drying (FD) has been used to dry high quality medical or recreational cannabis [11].

Curing is another important subsequent postharvest process of drying that significantly affects the quality of plant tissues, particularly the smokable inflorescence and leaves of tobacco [12]. Curing is sometimes called the ‘maturation’ or ‘aging’ process, which, in practice, is performed by storing the dried hemp in closed containers under specific temperature and humidity conditions for a certain period of time to allow for the change in flavor profiles [13]. This relatively long process under mild temperature and humidity allows moisture in the dried hemp to re-equilibrate to the controlled environmental conditions to mitigate any over-drying issues. Das et al. [4] claimed that the curing involves the enzymatic and bacterial breakdown of the sugars and starches created by chlorophyll breakdown. If not properly cured, these chemical compounds will result in rough, harsh-hitting smoke and a raw plant taste during burning. Jin et al. [13] suggested that curing at 18 °C and 60% RH for 2 weeks with the lid open after 6 h, rendered the best flower quality. Lazarjani et al. [14] stated that the curing process resulted in decarboxylation and an increase in THC and CBN in medicinal hemp flowers and increased potency. However, most published research on the hemp curing process are review articles, which provide limited amounts of scientific data on the quality attributes of the hemp that can verify the biochemical changes mentioned above. There is a critical need to generate experimental data related to curing conditions and product qualities (particularly related to the moisture, cannabinoids, color, and microbial levels), which will enhance the understanding of the curing process by researchers and industry stakeholders.

It was hypothesized that postharvest drying and curing could significantly affect the quality and safety attributes of hemp inflorescence. Therefore, the objective of this study is to generate scientific data about the hemp drying and curing processes from process engineering, chemical, and microbiological perspectives. This information will help hemp processors, distributors, and other stakeholders to adapt to emerging technologies for better quality and safety control of hemp products and reduce product loss due to poor postharvest practices.

## 2. Results and Discussion

### 2.1. Moisture Content and Drying Characteristics

The moisture content of the hemp floral biomass prior to drying, after drying, and after curing processes are summarized in Table 1. The initial moisture content of the Hempress and Wild Bourbon varieties ranged from 74.9% to 75.0%, and 77.4% to 80.6% on the wet basis, respectively. Following the different drying processes, the final moisture content ranged from 1.9% to 6.8% on the wet basis. The overall drying time was 8 h for HA drying, 48 h for FD drying, and 1 week for ambient air natural drying processes. The average drying rates ranged from 6.15 × 10^−^^3^ to 6.97 × 10^−^^3^ g moisture/g dry mass/min for HA drying, from 1.03 × 10^−^^3^ to 1.13 × 10^−^^3^ g moisture/g dry mass/min for FD drying, and from 0.29 × 10^−^^3^ to 0.35 × 10^−^^3^ g moisture/g dry mass/min for ambient air drying, respectively. The ambient air drying required the longest time to fully dry the hemp biomass, while HA drying needed the least time. The drying rates of HA drying were also ~5 times higher than the FD process. These results should be attributed to the different heat and mass transfer mechanisms during different drying processes. During the HA drying, the forced convective heat and mass transfer facilitates the rapid temperature increase in the hemp biomass and moisture evaporation from the sample surface [15]. During the ambient air-drying process, the low temperature gradient between the sample surface and the ambient air, as well as the slow air movement, made this natural convective drying process significantly less efficient than HA drying [16]. In contrast, the FD process occurred under high vacuum conditions, allowing water evaporation at much lower temperatures than under atmospheric conditions [17]. As a result, the drying rates of FD process were approximately three times higher compared to the ambient air-drying process, despite operating at lower temperature. These findings suggest that the HA drying method was much more efficient compared to the current ambient air-drying practice. Considering the availability of large-scale HA dryers for other agri-food products in the industry, this new drying technology can be easily translated into practice without excessive capital investment.

**Figure 1:**
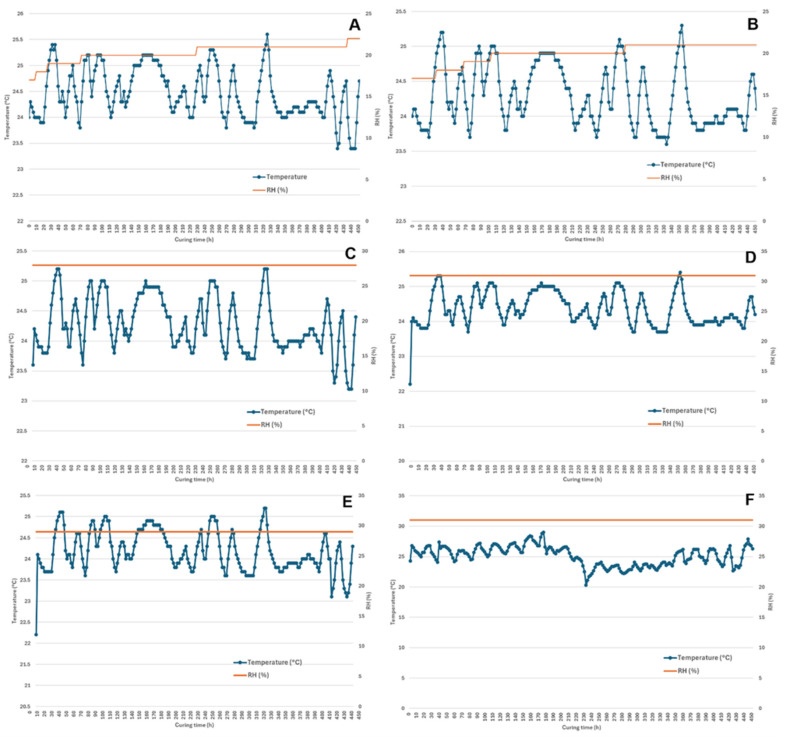
Average temperature and RH changes during the curing process: (**A**) FD in glass jar; (**B**) FD in Mylar bag; (**C**) Ambient air dried in glass jar; (**D**) Ambient air dried in Mylar bag; (**E**) HA in glass jar; (**F**) HA in Mylar bag.

After drying, the hemp biomass was packed into either Mylar bags or glass jars for the curing process. The environmental conditions inside the curing containers were recorded automatically. Figure 1 shows the typical temperature and RH curves for the Wild Bourbon cultivar under different drying and curing conditions. Meanwhile, the moisture content of the hemp biomass after curing are summarized in Table 1. In general, the curing process increased from 3.3% to 13.6% on the wet basis. The average room temperature and RH during the experiment was around 25 °C and 45%, respectively. For all the samples, the temperature remained relatively constant within the 24–25 °C range; the minor fluctuations should be attributed to the temperature changes during the day and night. The RH within the curing containers, on the other hand, varied among the specific samples. For FD samples, the RH increased from 17% to 22% over the 3-week curing process, resulting in the moisture content increasing from 6.1% to 11.5%, respectively, within the Mylar bags and glass jars. For ambient air-dried hemp at room temperature, the RH within the containers was 28% and 31%, respectively, for the Mylar bags and glass jars, which led to a decrease from 16.5% to 13.6% in moisture content. For HA dried samples, the moisture content increased from 5.8% to 12.1% and 15.8%, respectively, for Mylar bag and glass jar curing. The final moisture content of the hemp floral biomass after curing was influenced not only by the moisture content after drying, but also by the RH of the environment. Higher RH levels in the curing container resulted in higher final moisture content after curing. However, the influence of the curing container type on the final moisture content was not statistically significant. The increase in the hemp moisture content should be attributed to the slow moisture pick up by the dried hemp from the air to reach the equilibrium moisture content [18,19]. According to Oduola et al. [8], the equilibrium moisture content of the hemp biomass at 25 °C and within 15–45% RH range from 6.8% to 12.1% on the dry basis. The moisture content immediately after drying was lower than the equilibrium moisture content under the indoor curing conditions, which incurred the moisture absorption from the environment since equilibrium moisture content under the storage conditions was higher than that of the dried hemp.

### 2.2. Cannabinoids Content

**Figure 2:**
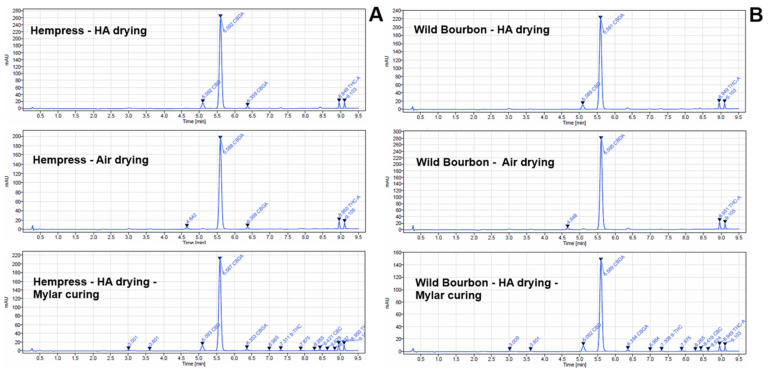
HPLC chromatography of the (**A**) Hempress cultivar and (**B**) Wild Bourbon cultivar hemp after HA drying, ambient air drying, and HA drying followed by curing in Mylar bag.

**Figure 3:**
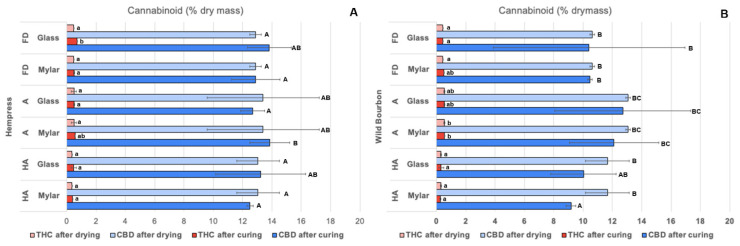
Total CBD and THC contents in the A) Hempress and B) Wild Bourbon hemp after drying and curing process; decarboxylation percentage and acid/neutral ratio. Same upper-case letters indicated that the mean values in the same column within the same cultivar were not significantly different (*p* > 0.01).

The typical HPLC chromatographs of the hemp biomass after selected drying and curing processes are shown in Figure 2. The retention times for CBD, CBDA, THCA, and THC were 4.64, 5.59, 8.95, and 9.11 min, respectively. Several of the other cannabinoids were also detected in the samples, but their concentrations were minor compared to the four major compounds. The total CBD content (calculated as the sum of CBD and CBDA derived CBD) in the dried hemp samples in this study was 13.04 ± 1.46, 13.40 ± 3.82, 12.88 ± 0.38 g/100 g of dried biomass under the ‘HA drying’, ‘Ambient drying’, and ‘Freeze drying’ of the Hempress cultivar, respectively; and was 11.66 ± 1.49, 13.06 ± 0.16, 10.62 ± 0.15 g/100 g dried for the Wild Bourbon cultivar (Figure 3). Similarly, the total THC content in the dried hemp samples was 0.37 ± 0.03, 0.50 ± 0.17, 0.46 ± 0.01, g/100 g of dried biomass under the ‘HA drying’, ‘Ambient drying’, and ‘Freeze drying’ of the Hempress cultivar, respectively, and 0.31 ± 0.04, 0.54 ± 0.03, 0.43 ± 0.01 g/100 g dried for the Wild Bourbon cultivar. The drying methods did not significantly influence the total cannabinoid contents (*p* > 0.01). Meanwhile, the ratio of the acidic to neutral form of cannabinoids under different drying and curing conditions is summarized in Table 2. In freshly harvested hemp, as well as in hemp dried by ambient air and freeze drying, neutral form cannabinoids (CBD, THC) were hardly detected. In contrast, they were present in samples dried by the HA method. This is because the relatively high temperatures during the HA drying process resulted in the decarboxylation of the acidic cannabinoids [20], a chemical reaction that eliminates the carboxyl group from the cannabinoid acid molecules and liberates carbon dioxide (CO_2_). The decarboxylation percentage for the Hempress and Wild Bourbon varieties were 9.0% and 8.5%, respectively. Compared to the stage right after the drying, the acid/neutral cannabinoids ratio reduced significantly, but the total cannabinoid content did not change. These results suggest that part of the acidic cannabinoids converted to their neutral forms during curing, likely due to the slow spontaneous biochemical reactions under mild temperature and humidity that led to decarboxylation under the mild curing conditions [21]. In general, the acid-to-neutral cannabinoid ratio of ambient air-dried hemp after curing process was lower than that from FD sample, suggesting that ambient air drying followed by curing resulted in more conversion of acidic cannabinoids to neutral forms. However, it should also be noted that the decarboxylation percentage was generally lower than 2%, except for samples subjected to HA drying. This should be due to the relatively low temperature and RH during the curing process, under which the decarboxylation was relatively low [22,23]. Better understanding of this process, e.g., the specific reaction mechanisms and kinetics during the hemp curing process, are needed for optimizing the process for the best product quality. Meanwhile, previous studies have shown that high temperature HA drying, microwave heating, or infrared heating could significantly promote cannabinoid decarboxylation, but also inactivate the enzymes in the biomass [8,10]. Therefore, identifying suitable postharvest drying and curing conditions for different types of hemp products is critical and requires further study.

### 2.3. Color Attributes

**Figure 4:**
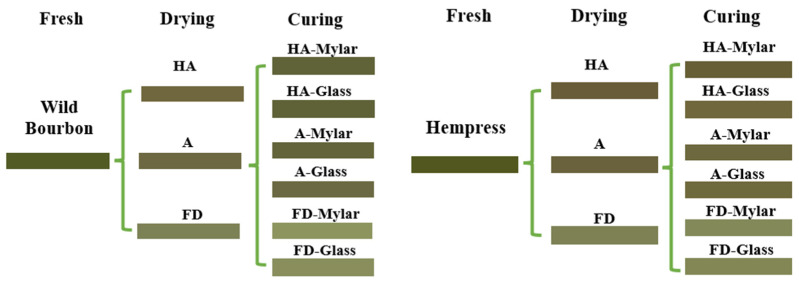
Color representation of the hemp after different drying and curing processes.

The color metrics of freshly harvested hemp biomass and after postharvest drying and curing are summarized in Table 3, with their color representation illustrated in Figure 4. In general, the drying process resulted in increases in the L* and a* and decreases in the b* values. The L* value represents the lightness (or brightness), which is a measure of the reflectance intensity of the solid sample surface upon illumination with a D65 flashlight. Since the dried tissue typically contains more void spaces in its microstructure that are filled with air than the fresh tissues, resulting in higher reflectance and brightness [24]. The highest and lowest L* values were found with FD hemp and HA hemp samples, while the lowest were found in HA hemp samples. This is because the FD plant materials usually have higher porosity with larger air pockets that are in favor of light reflection [25]; conversely, HA drying caused significant shrinkage of the solid microstructure, leaving less air pockets and lower reflectance intensity. After curing, the L* values increased slightly, but were not statistically significant (*p* > 0.01). The a* value measures the redness/greenness of the samples color. The negative a* values seen in Table 3 indicate that the color of the samples is green, with more negative a* values representing a greener color. For both varieties, drying caused the a* values to increase (less negative), suggesting that the reduction in greenness, which could be due to the degradation of the natural chlorophyll in the plant tissue during drying process [26]. Among the drying methods, HA drying led to the most decay of the greenness, while FD did not significantly increase the a* values. This was because the chlorophyll in the plant tissue was sensitive to the high temperature heating during the HA, while the low temperature FD protected this natural colorant. After curing, the a* values slightly reduced to more negative values, which was possibly due to the degradation of the natural anthocyanins [27]. However, such reductions were not statistically significant (*p* > 0.01). The b* values measure the blueness/yellowness of the plant tissue sample, with positive values representing yellow color, and negative values indicating blue colors. In general, the drying process resulted in significant reductions in the b* values, which could be attributed to the degradation of natural yellow colorants, such as carotenoids in the hemp flower [28]. The subsequent curing process led to slight increases in the b* values, but the changes were not statistically significant (*p* > 0.01).

The average color difference between different postharvest stages and the freshly harvested hemp biomass, along with the browning index, are also shown in Table 3. The most significant color change during the postharvest processes occurred during the drying. However, since the curing experiments in this study were only performed under indoor room conditions for only three weeks, further research should be performed under various strictly controlled conditions to better understand the changes to the hemp products during the long curing process.

### 2.4. Microbial Properties

**Figure 5:**
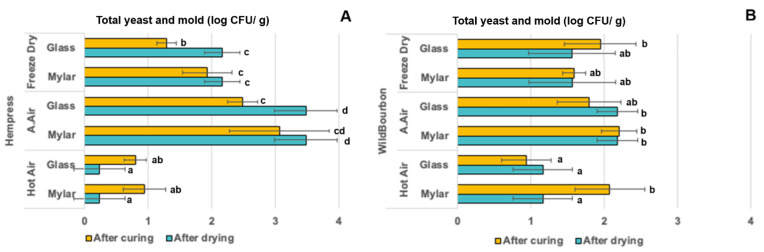
Total yeast and mold count on the hemp samples of (**A**) Hempress and (**B**) Wild Bourbon varieties after different drying and curing conditions. Same lower-case letters indicated that the mean values in the same column within the same cultivar were not significantly different (*p* > 0.01).

Plant tissues are highly susceptible to microbial contaminations by spoilage and pathogenic microorganisms. Previous studies have shown that industrial hemp can be infected by airborne bacteria, yeast, and mold during postharvest stages [29]. Particularly, cannabis are susceptible to Aspergillus contamination during postharvest, which may produce mycotoxins that threaten the safety of consumers. The rates of microorganism growth are primarily dependent on the environmental temperature and the amount of bioactive water within the tissue which is evaluated by water activity. In this study, total yeast and mold (TYM) count on the hemp samples after different postharvest processes were measured. For Hempress cultivar, the TYM levels after HA drying, ambient drying and FD ranged from 0.23 to 3.48 log CFU/g dry mass. After curing, the TYM levels ranged from 0.80 to 3.06 log CFU/g dry mass. HA dried samples had the lowest TYM level (up to 2 log lower than the other two drying methods), while ambient air-dried ones had the highest microbial loads. Except for the HA dried samples, the curing process reduced the TYM levels in the hemp samples; however, the reductions were not statistically significant. Furthermore, samples cured in Mylar bags had slightly higher TYM levels than those cured in glass jars, though the differences were not significantly different. For the Wild Bourbon cultivar, the TYM levels after HA drying, ambient drying, and FD ranged from 1.16 to 2.18 log CFU/g dry mass, respectively. After curing, the TYM levels ranged from 0.94 to 2.20 log CFU/g dry mass. Similar to the Hempress cultivar, the effects of curing on the change in TYM levels were not statistically significant. The results obtained in this study are consistent with previous reports that ambient air drying usually leads to microbial growth in the hemp biomass due to relative high-water activity during the initial stage of the drying. HA drying temperature could partly pasteurize the hemp biomass but is not considered adequate to ensure microbial safety. Results from Figure 5 and Figure 3 indicate that HA drying at 75 °C could reduce yeast and mold growth on hemp without compromising the cannabinoids content, which is different from previous reports by Chen et al. [10] that high temperature HA drying resulted in CBD loss. This difference may be attributed to the thermal sensitivity of different cultivars. Additionally, this study also shows that the curing process does not enhance the microbial safety risks of hemp by yeast and molds if the temperature and RH within the curing containers are properly controlled, which is the first time this has been reported. Findings from this study suggested that HA drying followed by glass jar curing is the preferred method for higher drying efficiency, better preservation of the cannabinoids, and enhanced microbial safety. Further research is still required to fill the knowledge gap in the understanding of the hemp curing process, which could include studying the curing influences on the terpene profiles, and kinetics of key physicochemical properties change, as well as carbohydrate compositions during the curing process.

## 3. Materials and Methods

### 3.1. Material Preparation

Two hemp varieties, Hempress and Wild Bourbon, available through the USDA-ARS National Plant Germplasm System (Accessions: G 33206 and G 33295), that are high in CBD and low in THC, were used in the experiments. For each variety, three plants were randomly selected, and 200 g samples were used. The ambient temperature and RH within the greenhouse ranged from 20–28 °C and 40–70%. Inflorescences of the hemp were manually harvested and trimmed from the Cornell University School of Integrated Plant Science greenhouse on 22 February 2024. The inflorescence of each cultivar was ground to 1 mm particle size and mixed thoroughly in a bag before being used for experiments.

The moisture content of the inflorescence was measured using a standard thermal gravimetric method for agricultural crops [30]. Specifically, 5 g hemp biomass was randomly selected from the bulk samples, weighed with an electronic balance, and then dried in an electrical oven at 105 °C for up to 24 h, or until the sample weight did not change significantly, and the moisture content was calculated using the following equations. The measurements were conducted in triplicate:

(1) $${MC}_{db}=\frac{\left(W_{w}-W_{d}\right)}{W_{d}}\;{MC}_{wb}=\frac{\left(W_{w}-W_{d}\right)}{W_{w}}$$

where MC_db_ and MC_wb_ are MCs of the hemp biomass sample on the dry basis (g moisture/g dry mass) and wet basis (g moisture/g wet mass) and W_w_ and W_d_ are wet and dry mass of the hemp biomass (g).

### 3.2. Chemicals and Reagents

Ultrapure water for high performance liquid chromatography (HPLC) analyses was obtained using a Milli-Q^®^ Integral 10 system (Millipore Sigma, Saint Louis, MI, USA). LC-grade chemicals for HPLC analyses, including formic acid, methanol, and ethanol were purchased from Fischer Scientific (Waltham, MA, USA). Standard LC-grade CBD cannabidiolic acid (CBDA), THC, tetrahydrocannabinolic acid (THCA), CBG, cannabigerolic acid (CBGA), cannabichromene (CBC), and cannabichromenic acid (CBCA) were purchased from Agilent (Santa Clara, CA, USA) for the preparation of calibration curves in HPLC analyses.

### 3.3. Postharvest Drying and Curing

**Figure 6:**
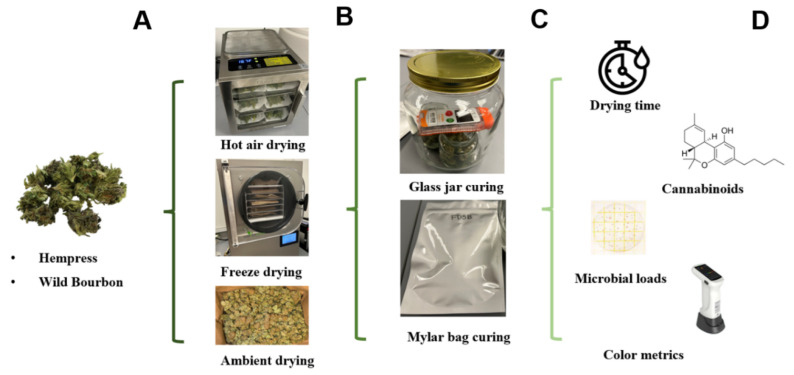
Experimental setup for hemp drying, curing, and properties analyses: (**A**) Freshly harvested hemp biomass; (**B**) Postharvest drying; (**C**) Postharvest curing; (**D**) Drying and quality analyses.

The research workflow showing the drying and curing experiment setup, microbial analysis, and cannabinoid and color analysis of the hemp biomass is shown in Figure 6. Freshly harvested and trimmed hemp biomass were dried using three methods: (1) Ambient air drying was conducted by placing the hemp biomass in paper bags and leaving in the lab with ventilation at 26 °C and 45% RH. (2) Hot air (HA) drying was performed in a lab-scale food dehydrator with digital temperature control (10CUDG, BenchFoods, Alvarado, TX, USA) at 75 °C. Fifty grams of samples were spread out in a thin layer on a piece of weighing paper and placed on stainless steel mesh trays. (3) Freeze drying (FD) was conducted in a bench-scale freeze dryer (Small Pro, HarvestRight, Salt Lake City, UT, USA). Specifically, 50 g of samples were packed in paper bags and placed within the freeze dryer at 2.1 kPa vacuum, a shelf temperature of 26 °C, and condenser temperature of −55 °C. Drying experiments were performed until the weight of the sample did not change. Weight change in the samples was measured with an electronic balance. The dryers were warmed up and ran for 30 min or until the desired drying conditions were achieved before experiments started. All drying experiments were performed in triplicate and the dried material was stored in sealed Ziploc bags in a −20 °C freezer for further analysis and curing experiments.

The drying time was defined as the time required to reduce the moisture content of samples from their initial levels to the end, then the average drying rates were calculated using Equation (2) [31]:

(2) $$\mathrm{DR}=\frac{{MC}_{0,db}-{MC}_{f,db}}{t_{d}}$$

where DR is drying rate (g moisture/g dry mass/min); MC_0,db_ and MC_f,db_ are initial and final moisture content of the samples (g moisture/g dry mass); and t_d_ is drying time (min).

The curing experiment of the dried hemp was performed in two types of containers: (1) Glass jars with lid sealed; (2) Mylar bags. The curing containers were placed in dark rooms in the same lab for 3 weeks. The temperature and RH within the containers during the curing process were monitored and recorded using a portable wireless sensor (M2H, Frigga, Shanghai, China). All curing experiments were performed with triplicate and the cured products were stored in sealed Ziploc bags in a −20 °C freezer for further analysis.

### 3.4. Color Measurement

The color metrics (in the coordinates of CIELab space, that is, L*, a*, and b*) of the fresh, dried, and cured hemp samples were measured using a CR-400 chromameter (Konica Minolta, Osaka, Japan). Each measurement was repeated six times, and the mean values were calculated.

The color difference in the samples after drying and curing compared to the freshly harvested hemp was calculated using Equation (3):

(3) $$\Delta E=\sqrt{{\Delta L}^{2}+{\Delta a}^{2}+{\Delta b}^{2}}$$

where ΔE is the color difference, and ΔL, Δa, and Δb represent the differences in the lightness, redness, and blueness, respectively.

The browning index (*BI*) of the samples after drying and curing processes is calculated using Equation (4) [32]:

(4) $$BI=\frac{\left[100\times (\frac{a+1.79\;L}{5.645\;L+a-3.012\;b}-0.31)\right]}{0.17}$$

### 3.5. Cannabinoid Content Measurement

The cannabinoid composition in the hemp samples at different stages of the postharvest were determined using a liquid chromatographic method. Specifically, approximately 100 mg of dried hemp inflorescence biomass under each condition was homogenized in 10 mL of HPLC grade methanol for 10 min to facilitate the extraction, using a VWR Vortexer 2 (Radnor, PA, USA) at room temperature. The samples were diluted 20-fold with methanol and filtered using a Captiva 0.45 μM regenerated cellulose filter before injecting 5.0 μL into the HPLC.

Samples were analyzed using an Agilent 1220 Infinity II LC system (Santa Clara, CA, USA) coupled with an Agilent Poroshell 120 column (2.7 μm, 3 × 50 mm, Santa Clara, CA, USA) following (Storm et al., 2018) [33]. The column was maintained at a constant temperature of 50 °C. The run began with an isocratic flow at 1 mL/min with a 60:40 ratio of methanol (containing 0.05% formic acid) to ultrapure water (containing 0.1% formic acid) for the first minute. This was followed by a 6-min gradient to 77% methanol and a subsequent 90 s gradient to 95% methanol. The concentrations of CBD, CBDA, THC, THCA, CBG, CBGA, CBC, and CBCA were determined based on UV absorbance at 230 nm.

The total potential cannabinoid content was calculated by summing the neutral and acidic forms, with adjustments for decarboxylation using conversion factors of 0.877 for THCA and CBDA and 0.878 for CBGA. The total CBD content in the samples was calculated based on the measured contents of CBDA and CBD as:

C_CBD,total_ = C_CBD_ + C_CBDA_ × 0.877 (5)

where C_CBD,total_, C_CBD_ and C_CBDA_ are the concentrations of total CBD, naturally existed CBD and CBDA in the sample (g CBD or g CBDA/100 g dried biomass), and 0.877 was the equivalent factor used to convert from CBDA contents to CBD contents.

The decarboxylation percentage (percentage of CBDA converted to CBD) during drying and curing processes was calculated with the equation below [10]:

(6) $$\mathrm{Conversion}=\frac{C_{CBD,drying}-C_{CBD,i}}{C_{CBDA,i}\times 0.877}\;\times \;100\%$$

where C_CBD,i_ and C_CBD_, drying are the concentrations of naturally existing CBD (g CBD/100 g dried biomass) in the fresh (represented by the freeze-dried sample) and dried samples and C_CBDA,i_ is the concentration of naturally existing CBDA (g CBDA/100 g dried biomass) in the fresh sample (represented by the freeze-dried sample).

The acid to neutral ratio of cannabinoids is calculated based on the relative contents of CBDA, CBD, THCA, and THC:

(7) $$\mathrm{Acid}\text{-}\mathrm{to}\text{-}\mathrm{neutral}\;\mathrm{ratio}=\frac{\left(\frac{C_{CBDA}}{C_{CBD}}+\frac{C_{THCA}}{C_{THC}}\right)}{2}$$

### 3.6. Microbial Level Measurement

Each sample was prepared by homogenizing 50 mg dried hemp flowers in 50 mL pure water for 30 min. The homogenized mixture was then diluted 5-fold with pure water. Subsequently, 500 μL of diluted mixture was spread out onto 3M PertriFilm^TM^ Rapid Yeast and Mold Count Plates (Charnwood Campus, Loughborough, UK) under biological hood, following the manufacturer’s instructions. These plates were incubated at room temperature (25–26 °C) for 72 h. The raw microbial loading counts were transformed using a base-10 logarithm function using the unit of CFU/g (Colony Forming Unit).

### 3.7. Statistical Analysis

Statistical analysis of the data was performed using SAS software (Version 9.4, SAS Institute Inc., Cary, NC, USA). Before the analyses, all data were screened for normality using the Shapiro–Wilk test. The data of sample moisture contents, cannabinoids contents, color metrics, and TAC levels were subjected to the analysis of variance (ANOVA) with post-hoc Tukey’s test (*p* < 0.01). Triplicate experiments were performed for all the tests.

## 4. Conclusions

HA drying of hemp inflorescence at 75 °C significantly increased the drying rates by up to 15 times compared to the conventional ambient air drying and reduced the total yeast and mold count by up to 2 log without significantly reducing the overall cannabinoids content. Meanwhile, HA drying resulted in partial decarboxylation of the cannabinoids with decreased acid-to-neutral ratio and greenness of the hemp. Curing in Mylar bags and glass jars under dark ambient conditions for 3 weeks led to an increase in the moisture content by 3.3% to 13.6%, which was dependent on the storage conditions. The slow curing process also resulted in slight decarboxylation but did not affect the cannabinoids content. Overall, the HA drying at 75 °C followed by curing in glass jars is a preferred method due to higher drying efficiency, better preservation of the cannabinoids, and improvement in microbial safety. Findings from this study provide fundamental information to fill in the knowledge gap in hemp postharvest, which is important for the industry to standardize the hemp processing to better product quality and safety management. Further research is needed to better understand the hemp curing process from engineering and biochemical perspectives.

## Figures and Tables

**Table 1:** Summary of moisture content at different stages of the hemp postharvest.

Cultivar	Drying Method	Curing Method	Initial Moisture	After Drying	After Curing
Hempress	Hot air	Mylar bag	75.0 ± 0.5% a	1.9 ± 0.9% a	10.2 ± 1.9% b *
		Glass jar			5.3 ± 2.8% a
	Ambient air	Mylar bag	75.0 ± 4.0% ab	6.8 ± 1.4% c	11.3 ± 2.7% b
		Glass jar			11.1 ± 1.9% b
	Freeze drying	Mylar bag	74.9 ± 0.6% a	4.0 ± 1.0% b	7.4 ± 3.2% a
		Glass jar			11.0 ± 2.4% b
Wild Bourbon	Hot air	Mylar bag	78.9 ± 2.1% b	5.8 ± 3.9% bc	12.1 ± 3.7% b
		Glass jar			15.8 ± 6.2% bc
	Ambient air	Mylar bag	77.4 ± 0.7% b	2.9 ± 3.3% a	16.5 ± 3.2% bc
		Glass jar			13.6 ± 5.4% b
	Freeze drying	Mylar bag	80.6 ± 4.0% b	2.8 ± 1.1% a	6.1 ± 5.2% a
		Glass jar			11.5 ± 5.2% b

* Same lower-case letters in the same column indicated that the mean values in the same column within the same cultivar were not significantly different (*p* > 0.01).

**Table 2:** Summary of acid to neutral cannabinoid ratio and decarboxylation percentage after different postharvest processing.

Cultivar	Status	Acid/Neutral Cannabinoid Ratio	Decarboxylation Percentage ^†^
	Initial	>100 *	/
	Drying		
	HA	13.7 ± 4.8	9.0% ± 3.0% c
	Ambient	>100	<0.1% a
	FD	>100	<0.1% a
	Curing		
Hempress	HA-Mylar	7.5 ± 0.8	9.2% ± 1.1% c
	A-Mylar	39.8 ± 3.4	1.6% ± 0.2% b
	FD-Mylar	60.3 ± 6.7	1.0% ± 0.1% b
	HA-Glass	7.9 ± 2.0	9.2% ± 2.7% c
	A- Glass	41.0 ± 2.4	1.5% ± 0.1% b
	FD- Glass	50.1 ± 9.9	1.3% ± 0.3% b
	Initial	>100	/
	Drying		
	HA	13.5 ± 1.1	8.5% ± 1.0% c
	Ambient	>100	<0.1% a
	FD	>100	<0.1% a
	Curing		
Wild Bourbon	HA-Mylar	8.4 ± 3.0	8.7% ± 3.0% c
	A-Mylar	35.9 ± 1.9	1.8% ± 0.1% b
	FD-Mylar	48.7 ± 3.5	1.3% ± 0.1% b
	HA-Glass	8.3 ± 1.3	8.3% ± 1.4% c
	A-Glass	47.3 ± 13.3	1.7% ± 0.2% b
	FD-Glass	26.3 ± 0.8	1.2% ± 0.6% b

* Note: >100 means the neutral cannabinoids were lower than detectable levels. ^†^ Decarboxylation percentage is calculated based on CBD and CBDA contents compared to the freshly harvested hemp; <0.1% means the neutral cannabinoids were lower than detectable levels; same lower-case letters indicated that the mean values in the same column were not significantly different (*p* > 0.01).

**Table 3:** Key color metrics of hemp after different drying and curing conditions.

Cultivar	Status	L*	a*	b*	ΔE	Browning Index
	Initial	35.55 ± 1.38 a	−10.88 ± 0.87 c	31.37 ± 0.80 c		
	Drying					
	HA	39.54 ± 2.49 b	−1.09 ± 1.44 a	22.93 ± 1.10 a	13.53 ± 1.55 a	85.49 ± 4.31 c
	Ambient	41.27 ± 2.25 b	−3.04 ± 0.95 a	22.67 ± 1.23 a	13.03 ± 1.42 a	75.41 ± 3.83 b
	FD	52.61 ± 4.29 c	−8.89 ± 1.47 c	23.77 ± 1.72 a	18.78 ± 2.33 b	49.36 ± 2.16 a
	Curing					
	HA-Mylar	39.71 ± 2.51 b	−2.23 ± 0.50 a	23.79 ± 0.89 a	12.23 ± 1.37 a	87.15 ± 5.22 c
Hempress	A-Mylar	43.57 ± 1.06 b	−4.78 ± 0.68 b	24.61 ± 0.82 ab	12.13 ± 0.87 a	75.45 ± 4.13 b
	FD-Mylar	55.70 ± 1.95 c	−9.71 ± 0.95 c	24.84 ± 0.70 ab	21.21 ± 1.05 b	47.91 ± 3.33 a
	HA-Glass	43.36 ± 1.51 b	−2.84 ± 0.50 a	25.27 ± 0.43 b	12.76 ± 0.88 a	82.92 ± 5.12 bc
	A- Glass	44.12 ± 0.43 b	−4.35 ± 0.35 b	24.89 ± 0.24 ab	12.57 ± 0.33 a	76.26 ± 3.77 b
	FD- Glass	54.91 ± 1.18 c	−10.21 ± 0.49 c	26.23 ± 0.51 b	20.04 ± 0.78 b	52.35 ± 3.01 a
	Initial	36.73 ± 2.44 A	−12.59 ± 1.63 D	30.42 ± 3.43 CD		
	Drying					
	HA	43.70 ± 1.92 B	−2.45 ± 1.20 A	24.02 ± 1.03 AB	13.87 ± 1.55 B	75.96 ± 4.43 C
	Ambient	43.48 ± 3.40 B	−3.83 ± 1.19 A	22.50 ± 1.65 A	13.60 ± 1.42 B	68.04 ± 2.73 C
	FD	52.65 ± 3.00 C	−8.73 ± 0.82 C	23.05 ± 1.21 A	17.96 ± 2.33 C	47.28 ± 3.16 A
Wild Bourbon	Curing					
	HA-Mylar	40.24 ± 0.63 B	−7.82 ± 0.51 C	23.79 ± 0.14 AB	8.90 ± 0.35 A	73.41 ± 5.12 C
	A-Mylar	41.39 ± 0.81 B	−6.65 ± 0.73 B	23.45 ± 0.32 A	10.28 ± 0.58 A	71.34 ± 5.73 C
	FD-Mylar	59.57 ± 0.95 D	−11.46 ± 0.20 D	28.07 ± 0.18 C	22.99 ± 0.53 D	50.65 ± 3.27 A
	HA-Glass	39.93 ± 1.17 AB	−7.93 ± 0.46 C	23.42 ± 0.24 AB	9.00 ± 0.75 A	72.04 ± 4.13 C
	A- Glass	43.77 ± 1.25 B	−6.09 ± 1.10 B	21.98 ± 0.61 A	12.77 ± 1.15 AB	60.83 ± 4.03 B
	FD- Glass	57.54 ± 1.23 D	−10.51 ± 0.66 D	26.00 ± 0.67 B	21.38 ± 0.80 CD	48.18 ± 2.41 A

* Same lower-case and upper-case letters indicated that the mean values in the same column within the same cultivar were not significantly different (*p* > 0.05).

## Data Availability

The raw data supporting the conclusions of this article will be made available by the authors on request.
